# Epigenetic age acceleration is associated with speed of pubertal growth but not age of pubertal onset

**DOI:** 10.1038/s41598-024-53508-z

**Published:** 2024-02-05

**Authors:** Catherine Kim, Kylie K. Harrall, Deborah H. Glueck, Christine Hockett, Dana Dabelea

**Affiliations:** 1 Departments of Medicine, Obstetrics & Gynecology, and Epidemiology, University of Michigan, 2800 Plymouth Road, Building 16, Room 405E, Ann Arbor, MI 48109 USA; 2 Lifecourse Epidemiology of Adiposity and Diabetes (LEAD) Center, University of Colorado Denver, Aurora, CO USA; 3 Department of Pediatrics, University of Colorado School of Medicine, Aurora, CO USA; 4 Department of Epidemiology, University of Colorado, Aurora, CO USA; 5 Avera Research Institute, Sioux Falls, SD USA; 6 Department of Pediatrics, University of South Dakota School of Medicine, Sioux Falls, SD USA

**Keywords:** Epidemiology, Paediatric research

## Abstract

Using data from a longitudinal cohort of children, we examined whether epigenetic age acceleration (EAA) was associated with pubertal growth and whether these associations were mediated by adiposity. We examined associations between EAA at approximately 10 years of age with pubertal growth metrics, including age at peak height velocity (PHV), PHV, and sex steroid levels and whether these associations were mediated by measures of adiposity including body mass index (BMI) and MRI-assessed visceral adipose tissue (VAT) and subcutaneous adipose tissue (SAT). Children (n = 135) with accelerated EAA had higher PHV (β 0.018, *p* = 0.0008) although the effect size was small. The association between EAA and age at PHV was not significant (β  − 0.0022, *p* = 0.067). Although EAA was associated with higher BMI (β 0.16, *p* = 0.0041), VAT (β 0.50, *p* = 0.037), and SAT (β 3.47, *p* = 0.0076), BMI and VAT did not mediate associations between EAA and PHV, while SAT explained 8.4% of the association. Boys with higher EAA had lower total testosterone (β  − 12.03, *p* = 0.0014), but associations between EAA and other sex steroids were not significant, and EAA was not associated with sex steroid levels in girls. We conclude that EAA did not have strong associations with either age at onset of puberty or pubertal growth speed, although associations with growth speed were statistically significant. Studies with larger sample sizes are needed to confirm this pattern of associations.

Puberty encompasses a wide range of physical transitions, most notably growth in stature and development of secondary sex characteristics. Genetic factors account for roughly half of phenotypic variation^1^. Increasing prevalence of overnutrition in early life, including in-utero, is associated with younger age at onset of puberty as well as speed of pubertal growth^2–5^. It is important to understand the pathways through which this occurs, since earlier sexual development is associated with earlier cardiometabolic dysregulation and other adverse outcomes^6–10^.

The epigenome refers to the modifications of the genome that may regulate gene expression and activity, but do not incorporate changes in the DNA sequence. The most commonly studied epigenetic modification is the addition of a methyl group to the cytosine-guanine pair; such methylation, particularly in close proximity to a gene, may reduce or silence gene expression^11^. Specific patterns of DNA methylation across the genome detected through epigenome wide association studies (EWAS) correspond with lifespan and mortality. These epigenetic “clocks” can be further compared against chronologic age, and the discrepancy between biologic age and chronologic age may be associated with future health outcomes^12^. Individuals with older estimates of biologic age compared to chronologic age are said to have epigenetic age acceleration (EAA), and larger degrees of EAA correspond with increased risk of dementia, mortality, and other conditions associated with older age^12^.

Most commonly, EAA in children has been examined with respect to stress and toxicants^13^, although a few studies have examined associations between EAA and growth indices in youth. In one cohort of Finnish children, children who had higher values for EAA also had greater height and weight and more advanced Tanner stage than children with lower values of EAA^14^. Another report has also noted that accelerated epigenetic aging was linked to higher body mass index (BMI)^15^. However, another cohort of English children noted no association between EAA at birth and future trajectories of height and weight^16^. Using data from the Exploring Perinatal Outcomes among Children Study (EPOCH), a longitudinal cohort study^17^, we have previously reported that greater EAA is associated with insulin resistance and insulin secretion in youth^18^.

Whether EAA is linked with pubertal growth indices is not known. Therefore, using data from the Exploring Perinatal Outcomes among Children Study (EPOCH), a longitudinal cohort study^19^, we examined whether higher EAA was linked with pubertal indices such as age at onset of the pubertal growth spurt indicated by age at peak height velocity or age at PHV, speed of the growth spurt or PHV, and other indicators of sexual development such as sex steroid levels. We also examined whether any associations were mediated through BMI and other measures of adiposity (Fig. 1, conceptual model).

**Figure 1:**
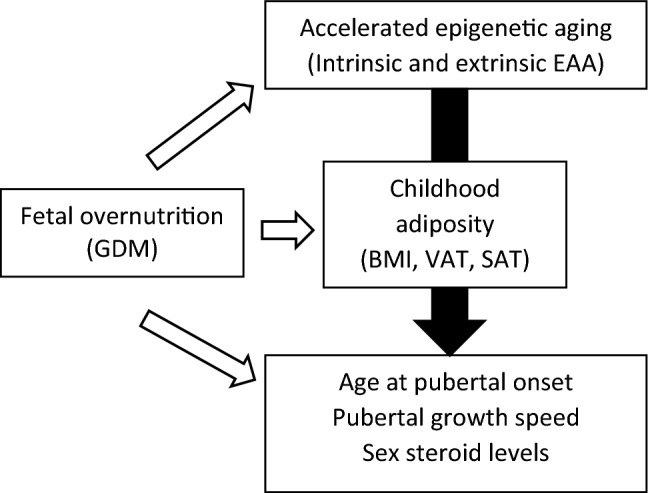
Conceptual model. Previous studies (white arrows) have demonstrated that fetal overnutrition, as represented by gestational diabetes mellitus (GDM), is associated with several offspring characteristics: accelerated epigenetic aging (as represented by higher values of EAA or epigenetic age acceleration); offspring adiposity (as represented by higher body mass index [BMI], visceral adipose tissue area [VAT], and subcutaneous adipose tissue area [SAT]); and indices of pubertal development (age at pubertal onset, pubertal growth speed, and sex steroid levels). The present analysis (black arrow) examines whether accelerated epigenetic aging is associated with indices of pubertal development and whether any associations were mediated by childhood adiposity.

## Results

Table 1 shows participant characteristics. Both boys and girls were approximately 10 years of age at visit 1 and 16 years of age at visit 2. Approximately half of the population was prepubertal at visit 1, and all of the children were Tanner stage 2 or beyond by visit 2. Although the average EAA was close to 0, indicating similar epigenetic age compared to chronologic age overall, the standard deviation was wide indicating a range of epigenetic ages compared to chronologic ages in the study sample. Less than half of the girls were pre-pubertal (Tanner stage 1) at the first EPOCH visit, and age at PHV was approximately 11 years of age. More than half of the boys were pre-pubertal at the first EPOCH visit, and the age at PHV was approximately 13 years of age. By visit 2, the age at which growth metrics and sex steroids were assessed, all children had started their pubertal transition.

**Table 1:** Participant characteristics (N = 135).

	Girls	Boys
	N = 68	N = 67
Age at visit 1 (years)	9.5 (1.5)	10.1 (1.5)
Age at visit 2 (years)	16.2 (1.3)	16.5 (1.2)
Race/ethnicity (n, %)		
Non-hispanic white	39 (57%)	49 (73%)
Hispanic	21 (31%)	16 (24%)
Non-hispanic black	4 (6%)	2 (3%)
Other	4 (6%)	0
Extrinsic epigenetic age acceleration at visit 1	− 0.35 (6.06)	0.02 (4.74)
Intrinsic epigenetic age acceleration at visit 1	0.004 (4.56)	− 0.03 (2.15)
Pre-pubertal (Tanner stage 1) at visit 1	31 (46%)	41 (62%)
Tanner stage 2 at visit 1	26 (38%)	20 (30%)
Tanner stage 3 at visit 1	7 (10%)	3 (5%)
Tanner stage 4 at visit 1	4 (6%)	2 (3%)
Tanner stage 5 at visit 1	0 (0%)	0 (0%)
Pre-pubertal (Tanner stage 1) at visit 2	0 (0%)	0 (0%)
Tanner stage 2 at visit 2	0 (0%)	0 (0%)
Tanner stage 3 at visit 2	8 (12%)	2 (3%)
Tanner stage 4 at visit 2	32 (47%)	25 (37%)
Tanner stage 5 at visit 2	28 (41%)	40 (60%)
Age at peak height velocity (years)	11.3 (1.0)	13.1 (1.1)
Peak height velocity (cm/year)	8.4 (0.4)	9.5 (0.2)
Body mass index Z-score at visit 1	0.2 (1.1)	0.2 (1.4)
Body mass index at visit 1 (kg/m^2^)	17.9 (3.4)	18.6 (4.1)
Visceral adipose tissue at visit 1 (cm^2^)	20.6 (14.7)	24.4 (19.4)
Subcutaneous adipose tissue at visit 1 (cm^2^)	103.4 (70.2)	104.7 (93.7)
Dehydroepiandrosterone at visit 2 (ng/dl)	157.2 (83.3)	226.7 (110.8)
Testosterone at visit 2 (ng/dl)	38.7 (16.4)	476.0 (147.7)
Estradiol at visit 2 (pg/ml)	78.8 (77.1)	25.1 (10.8)

Means (standard deviations) or n (percent) shown.

Table 2 shows associations between EAA with pubertal growth. Higher values of extrinsic EAA (indicating older epigenetic age in relation to chronologic age) were associated with a slightly higher PHV or pubertal growth spurt. However, the association between EAA and age at PHV did not meet statistical significance, although the sample size may not have been large enough to detect associations. Higher values of extrinsic EAA were also associated with greater body mass and adiposity indices as reflected by BMI, visceral adipose tissue (VAT), and subcutaneous adipose tissue (SAT) at visit 1. Higher extrinsic EAA at visit 1 was also associated with lower testosterone levels at visit 2 in boys but not in girls. Intrinsic EAA was not associated with measures of pubertal growth, adiposity, or sex steroid levels.

**Table 2:** Association between epigenetic age acceleration (EAA) (independent variable) with dependent variables of growth metrics, sex steroid levels at visit 2, and adiposity measures at visit 1.

	Extrinsic EAA	Intrinsic EAA
Peak height velocity (cm/year)	**0.018 (0.008, 0.028)**	0.011 (− 0.006, 0.027)
	***p* = 0.0008**	*p* = 0.22
Age at peak height velocity (years)	− 0.0022 (− 0.0046, 0.0002)	− 0.0029 (− 0.0066, 0.0008)
	*p* = 0.067	*p* = 0.12
Testosterone (ng/dl) at visit 2		
Boys	− **12.03 (**− **19.21, ** − **4.85)**	2.58 (− 16.04, 21.20)
	***p* = 0.0014**	*p* = 0.78
Girls	0.24 (− 0.55, 1.03)	0.068 (− 1.10, 1.24)
	*p* = 0.55	*p* = 0.91
Dehydroepiandrosterone (ng/dl) at visit 2	1.19 (− 1.92, 4.30)	2.75 (− 1.29, 6.79)
	*p* = 0.45	*p* = 0.18
Estradiol (pg/ml) at visit 2	0.049 (− 1.71, 1.81)	1.26 (− 1.66, 4.18)
	*p* = 0.96	*p* = 0.40
Body mass index (kg/m^2^) at visit 1	**0.16 (0.050, 0.27)**	0.096 (− 0.096, 0.29)
	***p* = 0.0044**	*p* = 0.32
Visceral adipose tissue (cm^2^) at visit 1	**0.48 (0.020, 0.95)**	0.27 (− 0.52, 1.06)
	***p* = 0.041**	*p* = 0.49
Subcutaneous adipose tissue (cm^2^) at visit 1	**3.50 (0.96, 6.03)**	1.92 (− 2.44, 6.28)
	***p* = 0.0071**	*p* = 0.39

Models adjust for race and ethnicity, as well as sex unless otherwise indicated. Beta-coefficients, 95% confidence intervals, and *p*-values are shown.

Significant values are in bold.

We conducted several sensitivity analyses. First, we examined whether EAA was still associated with the speed of pubertal growth even after adjustment for chronologic age. In models that examined whether EAA was associated with pubertal growth speed after adjustment for chronologic age and epigenetic age acceleration in the same model, EAA still was associated with PHV (beta coefficient 0.018, 95% CI 0.0076, 0.028) but chronologic age was not (beta coefficient  − 0.033, 95% CI  − 0.070, 0.0046). This suggests that an individual child’s epigenetic estimates of age regressed on their chronologic age provided additional information apart from chronologic age alone. Second, we examined whether EAA might be associated with the speed of pubertal growth through the associations with adiposity. We conducted mediation testing^20,21^, where we examined whether we calculated the proportion of the association between EAA and PHV was mediated by each measure of adiposity (Table 3). In these models, BMI explained only 0.6% of the association between extrinsic EAA and PHV, VAT explained only 1.9% of the association, and SAT explained 8.4% of the association. We conducted similar testing for the association between extrinsic EAA and testosterone in boys (Table 4). Adiposity did appear to play a more significant role in mediating the associations between extrinsic EAA and lower testosterone in boys, although not for the majority of the association: BMI explained 13.8% of the association between extrinsic EAA and testosterone, VAT explained 10.6% of the association, and SAT explained 11.2% of the association.

**Table 3:** The mediating role of adiposity measures between extrinsic epigenetic age acceleration and peak height velocity.

Adiposity measure	Effect	Estimate	95% CI	Proportion mediated
Body mass index (kg/m^2^) at visit 1	Total effect	0.018	0.0080, 0.028	0.6%
	Natural direct effect	0.017	0.0068, 0.028	
	Natural indirect effect	0.0011	− 0.0014, 0.0036	
Visceral adipose tissue (cm^2^) at visit 1	Total effect	0.018	0.0073, 0.028	1.9%
	Natural direct effect	0.018	0.0069, 0.028	
	Natural indirect effect	0.00034	− 0.0013, 0.0020	
Subcutaneous adipose tissue (cm^2^) at visit 1	Total effect	0.018	0.0077, 0.029	8.4%
	Natural direct effect	0.017	0.0061, 0.027	
	Natural indirect effect	0.0015	− 0.0012, 0.0042

**Table 4:** The mediating role of adiposity measures between extrinsic epigenetic age acceleration and testosterone levels in boys.

Adiposity measure	Effect	Estimate	95% CI	Proportion mediated
Body mass index (kg/m^2^) at visit 1	Total effect	− 11.29	− 18.48, − 4.10	13.8%
	Natural direct effect	− 9.74	− 17.66, − 1.81	
	Natural indirect effect	− 1.56	− 4.74, 1.63	
Visceral adipose tissue (cm^2^) at visit 1	Total effect	− 11.04	− 18.50, − 3.58	10.6%
	Natural direct effect	− 9.87	− 17.49, − 2.25	
	Natural indirect effect	− 1.18	− 3.15, 0.80	
Subcutaneous adipose tissue (cm^2^) at visit 1	Total effect	− 10.99	− 18.49, − 3.49	11.2%
	Natural direct effect	− 9.76	− 17.93, − 1.60	
	Natural indirect effect	− 1.23	− 4.40, 1.94	

## Discussion

Using data from a longitudinal cohort study that characterized the pubertal growth spurt, MRI-assessed adiposity, and sex steroids, we found that higher extrinsic EAA was associated with a faster pubertal growth spurt. This association persisted even after additional adjustment for chronologic age, indicating that extrinsic EAA provides additional information regarding future growth even after considering a child’s age. Higher extrinsic EAA was also associated with higher BMI, greater VAT, and greater SAT. Although higher extrinsic EAA was associated with BMI, VAT, and SAT and thus a marker for growth speed, it was not associated with actual age at PHV, suggesting that EAA may not be involved in the onset of pubertal development. However, the magnitude of the association between EAA and growth speed was small, suggesting that the relationship between EAA and this particular pubertal index is unlikely to explain previously reported associations between adverse childhood exposures and pubertal development.

The study of EAA in humans has focused upon mortality and disease outcomes, particularly in adults. A growing body of evidence suggests that such indicators of more rapid epigenetic aging may also be associated with developmental measures in children. Youth with greater EAA are more likely to have experienced stress/adverse childhood experiences^22^ with future cognitive impairment^23^ and depression and anxiety^24^. Our report adds to the growing body of work that examines EAA with respect to childhood markers of growth and maturation. Our results are congruent with a previous study examining the relationship between EAA and childhood growth^14^. In a cohort of Finnish children, higher intrinsic EAA at age 7 years was associated with later pubertal stage^14^, suggesting that EAA could be a pathway through which overnutrition either in-utero or in early childhood might speed growth and development. Our report adds to that study by our examination of a racially diverse group of children, as well as our examination of EAA at 11 years of age, closer to the typical growth spurt characterizing puberty. We also examined additional metrics including sex steroid levels, MRI assessments of fat in addition to body mass index, height, and weight, and extrinsic EAA as well as intrinsic EAA. In contrast, higher EAA at birth was not associated with trajectories of height by ten years of age in a cohort of English youth^16^ although it was associated with lower weight at ten years of age. The different pattern of associations may have been due to their assessment of EAA at birth, as opposed to early childhood, or examination of growth prior to the pubertal growth spurt.

We did not find associations between EAA and age of onset, suggesting that EAA may have slightly stronger relationships with speed of growth rather than triggering pubertal maturation. It is possible that an association may have been detected with larger sample sizes. The age of onset and speed of the pubertal growth spurt are typically correlated, with early developers also having higher PHV, although correlation is imperfect; in one analysis, the correlation was 0.45 in boys and 0.31 in girls^25^. Our results do not exclude smaller estimates of association.

We also found that EAA was associated with greater fat measures, congruent with prior reports^26^. However, these fat measures were not the primary pathway between EAA and PHV. Therefore, the relationship between adiposity and earlier onset of puberty does not involve EAA. Although adiposity has long been recognized as an important determinant of the pubertal growth spurt and sex steroid levels in multiple populations^27–32^, it is not the only factor associated with growth and development. Even in young children, EAA may represent a cumulative sum of exposures, including but not limited to overnutrition. Among children enrolled in the Human Early-Life Exposome project, exposures including maternal smoking during pregnancy and indoor particulate matter absorbance were linked with greater EAA at the age of 7 years^33^. Others have reported that adverse childhood experiences have been linked with specific methylation signatures^34,35^. Interventions aimed at slowing epigenetic aging are primarily being tested in animal models, although randomized trials of lifestyle modification in older adults results in slower EAA^36^. Whether epigenetic aging can or should be modified in children is not established.

We also found that higher extrinsic EAA was associated with lower testosterone levels in boys. As with the association between EAA and PHV, the effect size was small. Sex steroid levels in adolescence reflect multiple factors, including but not only pubertal timing. To some extent, this reflects the fact that greater fat mass is associated with lower testosterone in boys, since this association was partially mediated by the various fat measures available in EPOCH. In previous EPOCH reports^19,37^, more rapid accumulation of VAT or SAT was associated with lower testosterone independent of confounders including age, insulin and leptin levels.

We found a significant pattern of associations using extrinsic EAA rather than intrinsic EAA. This may be due to the fact that EPOCH estimates of EAA were derived from blood, and the Hannum calculator (the basis of extrinsic EAA) was trained using blood, whereas the Horvath calculator (the basis of intrinsic EAA) was trained using a range of tissue types. Which epigenetic calculator has the strongest associations with developmental milestones in children is not established, and EAA clocks developed in youth may perform better than those including older individuals^38^. However, despite these differences in associations, and the fact that these clocks share only 6 CpG sites of the 71 CpG sites in the Hannum clock^39^ and the 353 CpG sites in the Horvath clock^40,41^, intrinsic and extrinsic EAA estimates are highly correlated (r = 0.76) with each other^42^. An analysis of microarray expression data from monocytes note that these different clocks do share several overlapping transcriptional profiles, namely involving epidermal growth factor receptor signaling, mitochondrial translation and function, and oxidative phosphorylation, whereas transcriptional profiles unique to each clock are hypothesized to reflect tissue differences reflecting how each clock was developed^43^.

The strengths of this report include a diverse longitudinal cohort and multiple growth metrics, particularly for adiposity. Few studies have examined the relationship between EAA and speed of growth. However, there are several limitations. As EPOCH is an observational study, it cannot prove causality, and our findings are exploratory. EPOCH only assessed EWAS at one point in time, in pre-puberty, and repeated measures would be useful in determining directionality of associations. We were unable to control for genetic factors associated with pubertal timing and growth. Finally, our sample size was small, and we conducted multiple comparisons, raising the possibility that some of the associations might be due to chance.

We conclude that extrinsic EAA was associated with PHV but not age at PHV in a racially and ethnically diverse sample of youth, and PHV was also associated with lower testosterone levels in boys. Although children with EAA also had greater body mass and fat mass as represented by BMI, VAT, and SAT, adiposity was not the primary pathway between EAA and PHV. Our findings suggest that EAA and adiposity may be associated with PHV through different pathways. Future studies should replicate these findings in larger samples and at multiple points in time. As the number of EWAS studies among children expands, EAA might be a useful representation of childhood development due to its correlation with multiple markers to growth and development.

## Methods

EPOCH is an observational historical prospective study that recruited healthy 6- to 13-yr old children who were offspring of singleton pregnancies, born at a single hospital in Colorado between 1992 and 2002, whose biological mothers were members of a managed care plan in Colorado^44^. The study population was sampled to reflect similar racial and ethnic distributions of Colorado^45^. The first research visit (visit 1) occurred at a mean (SD) age of 10.4 (1.5) years, and the second research visit (visit 2) occurred at a mean (SD) age of 16.7 (1.2) years. All participants provided informed consent, and youths provided written assent. The study was approved by the Colorado Multiple Institutional Review Board, and all methods were performed in accordance with the relevant guidelines and regulations. The original goal of the EPOCH cohort was to determine how exposure to in-utero overnutrition subsequently influenced trajectories of childhood adiposity and growth. Children were excluded if they had been exposed to intrauterine growth restriction (defined as birthweight for gestational age score < 10% percentile). In-utero overnutrition was represented as maternal gestational diabetes mellitus (GDM) and the cohort had a case–control design which matched by GDM status. Among the subset who underwent EWAS, approximately one-half of children had mothers with GDM and half did not. Mothers with and without GDM had similar levels of education (approximately 83% with at least some postsecondary education) and income (approximately 45% with a household income > $50,000 per year)^17^. Approximately 5% had preeclampsia, which did not differ by GDM history. Although maternal smoking was recorded (approximately 15% of mothers with GDM compared to 5% of mothers without GDM), exposure to second-hand smoke was not.

Conduction of EWAS in EPOCH has been previously described^46^. Using samples from visit 1, DNA was isolated from buffy coats that had been stored at  − 80 °C using the QIAamp kit (Qiagen, Germantown, MD). DNA samples were quantified, and purity was assessed using a NanoDrop spectrophotometer and a Qubit fluorometer (Thermo Scientific, Wilmington, DE). We measured genome-wide DNA methylation using Illumina’s Infinium Human Methylation 450 k BeadChip on bisulfite-treated samples, and used standard quality control procedures^46^. To account for cell composition variability we estimated the proportions of CD4 + T lymphocytes, CD8 + T lymphocytes, B lymphocytes, natural killer cells, monocytes, and granulocytes using the Houseman et al.^47^ method.

Multiple calculators exist for epigenetic age; for the purposes of this report, we chose to examine 2 calculators based upon previous investigations in EPOCH^18^ and other cohorts^48^ or puberty^14^. Next, we calculated “extrinsic” epigenetic age acceleration (EAA) using standard approaches^40,42^. Briefly, Hannum epigenetic age was combined with three imputed blood cell components (naïve cytotoxic T cells, exhausted cytotoxic T cells, and plasmablasts) to form an aggregate measure. This measure was regressed onto chronological age, with the residual being extrinsic EAA. Thus, extrinsic EAA captures both epigenetic age as well as the weighted average of age-related characteristic changes in blood cell composition. A positive value indicates higher epigenetic age compared to chronologic age, whereas a negative value indicates lower epigenetic age compared to chronologic age. Finally, we calculated “intrinsic” EAA using standard approaches^40,42^. Briefly, Horvath epigenetic age was regressed onto chronological age, with the residual being intrinsic EAA^40,42^.

Pubertal staging may be represented through growth metrics or secondary sex characteristics such as menarche, pubarche, thelarche, and gonadarche. Growth metrics may be less subject to inter-observer biases than staging using sexual characteristics^49^. Although growth metrics are often not available due to the necessity of multiple visits, EPOCH participants had multiple (mean 19 visits) heights available from medical records^5^. The Superimposition by Translation and Rotation (SITAR) growth curve analysis uses a shape invariant spline curve and a nonlinear random-effects model to estimate an average growth curve for the entire sample and each individual’s deviation from this average curve^50^. Velocity or PHV estimates how the child’s growth velocity is compared with the average population velocity. The models were fitted with the SITAR package in R (sitar 1.0.8). To exclude the postnatal growth spurt, only heights and weights after 2 years of age were included.

Puberty is also characterized by increased adrenal production of dehydroepiandrosterone (DHEA) followed by gonadal production of serum testosterone and estradiol (E2)^49^. In EPOCH, fasting blood draw between 7 and 10 AM occurred for all consenting children at both the first and second visits. Sera from the second research visit were refrigerated and analyzed within 24 h of collection. All laboratory measurements were performed at the Colorado Clinical Translational Science Institute Core Laboratories. Serum DHEA was measured by using a Beckman Coulter chemiluminescent assay with a limit of detection of 0.05 micromol/L. Serum total testosterone was measured by using a Beckman Coulter 1‐step competitive with a limit of detection of 0.59 nmol/L. Serum E2 was measured by using a Beckman Coulter chemiluminescent with a limit of detection of 36.7 pmol/L.

Childhood height and weight were measured in light clothing and without shoes. Weight was measured to the nearest 0.1 kg using an electronic scale. Height was measured to the nearest 0.1 cm using a portable stadiometer. Body mass index (BMI) was calculated as kg/m^2^. BMI Z-score was determined using the Centers for Disease Control and Prevention 2000 growth charts. Waist circumference was measured according to the National Health and Nutrition Examination Survey protocol as previously described^51^. Abdominal magnetic resonance imaging (MRI) was used to quantify VAT and SAT with a 3 T HDx Imager (General Electric, Waukashau, WI, USA) by a trained technician. Each study participant was placed supine and a series of T1-weighted coronal images were taken to locate the L4/L5 plane. One axial, 10 mm, T1-weighted image, at the umbilicus or L4/L5 vertebrae, was analyzed to determine SAT and VAT content. Images were analyzed by a single reader, blinded to exposure status. Previous reports in the EPOCH cohort have examined the correlation between BMI and specific markers of visceral adiposity. BMI has high correlation with SAT in boys (Pearson’s r = 0.94) and girls (r = 0.91), but the correlation with VAT is moderate (r = 0.65 in boys, r = 0.68 in girls)^51^. For the purposes of these analyses, measures of BMI, SAT, and VAT were examined from visit 1.

Demographic information (age, sex, and race/ethnicity) were collected via self‐report. Race/ethnicity was collected using 2000 U.S. Census-based questions and categorized as Hispanic (any race), non-Hispanic white, non-Hispanic Black, and non-Hispanic other. Pubertal development was self-reported by the offspring at each visit using diagrammatic representations of Tanner staging adapted from Marshall and Tanner^52^. Pubertal stage at each research visit was ascertained by child’s self-report, which has been noted to have excellent agreement with physician-assessed Tanner stage^53^. Tanner stage 1–5 was classified on the basis of the appearance of pubic hair for males and the stage of breast development for females. We further categorized puberty into pre-pubertal (Tanner stage 1) and pubertal and post-pubertal (Tanner stage 2–5).

### Statistical analysis

The present analysis focuses upon the children who underwent EWAS at visit 1 (n = 179) and who had electronic medical data allowing for calculation of growth metrics, for a total of 135 children. The sample size was determined by the number of EPOCH participants who had undergone EWAS and also had PHV measurements available (i.e. had height measurements enabling SITAR growth curve analysis). Based on a previous report by Suarez et al.^14^, we estimated that this would likely be large enough to detect statistically significant associations, specifically 0.018/cm/year in PHV.

Baseline characteristics were described using numbers (percentages) for categorical variables and means (standard deviations) for quantitative variables (Table 1). Two statistical models were used to examine relationships between EAA, growth metrics, and sex steroids. General linear models were fit for continuous pubertal outcomes including PHV and sex steroid levels (DHEA, testosterone, and E2) measured at visit 2. Jackknife residuals were used to assess model assumptions. Separate models were fit to assess the association, or total effect, between each main independent variable and pubertal outcome. Main independent variables included intrinsic EAA and extrinsic EAA. All models were adjusted for sex, an indicator variable for non-Hispanic white, an indicator variable for Hispanic, and the two-way interaction between sex and the main independent variable. If the sex interaction was not significant, it was removed from the model. Associations between EAA and sex hormones were not significant with the exception of testosterone, and thus models of EAA and testosterone are presented stratified by sex.

When we examined the association between EAA and age at PHV, we fit accelerated failure time models with a log-logistic distribution for the time-to-event outcome. The log-logistic distribution allows for a non-monotonic hazard function, allowing the hazard to increase or decrease over time. There was no censoring, because all participants were observed at the age at which PHV occurred. Log-logistic probability plots were used to assess model assumptions. Hypothesis tests were conducted using the Wald chi-square test and F-tests. Significance was determined using *p* < 0.05 for main effects and *p* < 0.10 for interactions. Analyses were performed in the statistical analysis software (SAS) version 9.4 (SAS Institute).

Mediation hypotheses were tested using methods of Valeri and VanderWeele^21,54^ which accommodate both accelerated time failure models and general linear models. Mediation hypotheses examined whether childhood adiposity (visit 1 BMI, VAT, or SAT) mediated the association between EAA and pubertal outcomes (age at PHV, PHV, sex steroid levels) (Fig. 1). Two models were fit for each hypothesis and mediator. First, the best-fitting model of the total effect was further adjusted for the mediator and the two-way interaction between the mediator and the main independent variable. If the interaction was non-significant, it was removed from the model. Second, a model was fit to assess the association between the main independent variable and the mediator. This model was also adjusted for sex and race/ethnicity. The total effect of the first model was then decomposed into the natural direct effect, the natural indirect effect, and the proportion mediated using the second model. Accelerated failure time models were applied as this was the methodology that EPOCH had previously reported for examining the relationship between other exposures with age at PHV^5^. However, when we examined residuals in our mediation analyses, general linear models had residuals that fit the model assumptions as compared to accelerated time failure models. The pattern of association was similar between accelerated time failure models and general linear models. Therefore, general linear models were used for these mediation analyses.

## Data Availability

The datasets analyzed during the current study are available from the corresponding author on reasonable request.

## References

[1] Towne B (2005). Heritability of age at menarche in girls from the Fels Longitudinal Study. Am. J. Phys. Anthropol..

[2] Chen L (2021). Trajectory of BMI from ages 2 to 7 years and age at peak height velocity in boys and girls. J. Pediatr..

[3] Huang A, Roth C (2021). The link between obesity and puberty: What is new?. Curr. Opin. Pediatr..

[4] Aris I (2022). Analysis of early-life growth and age at pubertal onset in U.S. children. JAMA Netw. Open.

[5] Hockett C (2019). Exposure to diabetes in utero is associated with earlier pubertal timing and faster pubertal growth in the offspring: The EPOCH study. J. Pediatr..

[6] Dreyfus J (2015). Age at menarche and cardiometabolic risk in adulthood: The coronary artery risk development in Young Adults Study. J. Pediatr..

[7] Lakshman R (2009). Early age at menarche associated with cardiovascular disease and mortality. J. Clin. Endocrinol. Metab..

[8] Lee J (2019). Age at menarche and risk of cardiovascular disease outcomes: Findings from the national heart lung and blood institute-sponsored women's ischemia syndrome evaluation. J. Am. Heart Assoc..

[9] Canoy D (2014). Age at menarche and risks of coronary heart and other vascular diseases in a large UK cohort. Circulation.

[10] Chen X (2018). Age at menarche and risk of all-cause and cardiovascular mortality: Systematic review and dose-response meta-analysis. Menopause.

[11] Egger G, Liang G, Aparicio A, Jones PA (2004). Epigenetics in human disease and prospects for epigenetic therapy. Nature.

[12] Faul JD (2023). Epigenetic-based age acceleration in a representative sample of older Americans: Associations with aging-related morbidity and mortality. Proc. Natl. Acad. Sci. USA.

[13] Nwanaji-Enwerem JC (2021). Maternal adverse childhood experiences before pregnancy are associated with epigenetic aging changes in their children. Aging.

[14] Suarez A (2018). The epigenetic clock and pubertal, neuroendocrine, psychiatric, and cognitive outcomes in adolescents. Clin. Epigenetics.

[15] Etzel L (2022). Obesity and accelerated epigenetic aging in a high-risk cohort of children. Sci. Rep..

[16] Bright H (2019). Epigenetic gestational age and trajectories of weight and height during childhood: A prospective cohort study. Clin. Epigenetics.

[17] Hockett CW, Harrall KK, Glueck DH, Dabelea DM (2023). Exposure to gestational diabetes and BMI trajectories through adolescence: The exploring perinatal outcomes among children study. J. Clin. Endocrinol. Metab..

[18] Kim C, Harrall K, Glueck D, Needham B, Dabelea D (2022). Gestational diabetes mellitus, epigenetic age, and offspring metabolism. Diabet. Med..

[19] Kim C, Harrall K, Glueck D, Shumer D, Dabelea D (2019). Childhood adiposity and adolescent sex steroids in the exploring perinatal outcomes among children study. Clin. Endocrinol..

[20] Valeri L, Vanderweele T (2013). Mediation analyses allowing for exposure-mediator interactions and causal interpretation: Theoretical assumptions and implementation with SAS and SPSS macros. Psychol. Methods.

[21] Valeri L, VanderWeele T (2015). SAS macro for causal mediation analysis with survival data. Epidemiology.

[22] Joshi D, Gonzalez A, Lin D, Raina P (2023). The association between adverse childhood experiences and epigenetic age acceleration in the Canadian longitudinal study on aging (CLSA). Aging Cell.

[23] Felt JM (2023). Epigenetic age acceleration as a biomarker for impaired cognitive abilities in adulthood following early life adversity and psychiatric disorders. Neurobiol. Stress.

[24] Zhang ZZ (2023). The association of epigenetic age acceleration and depressive and anxiety symptom severity among children recently exposed to substantiated maltreatment. J. Psychiatr. Res..

[25] Areekal SA, Goel P, Khadilkar A, Khadilkar V, Cole TJ (2022). Assessment of height growth in Indian children using growth centiles and growth curves. Ann. Hum. Biol..

[26] Etzel L (2022). Obesity and accelerated epigenetic aging in a high-risk cohort of children. Sci. Rep..

[27] O'Keefe L, Frysz M, Bell J, Howe L, Fraser A (2020). Puberty timing and adiposity change across childhood and adolescence: Disentangling cause and consequence. Hum. Reprod..

[28] Li Y (2022). Adiposity status, trajectories and the earlier puberty onset: Results from a longitudinal cohort study. J. Clin. Endocrinol. Metab..

[29] Pereira A (2021). Total and central adiposity are associated with age at gonadarche and incidence of precocious gonadarche in boys. J. Clin. Endocrinol. Metab..

[30] Buyken A, Karaolis-Danckert N, Remer T (2009). Association of prepubertal body composition in healthy girls and boys with the timing of early and late pubertal markers. Am. J. Clin. Nutr..

[31] Busch A, Hojgaard B, Hagen C, Teilmann G (2020). Obesity is associated with earlier pubertal onset in boys. J. Clin. Endocrinol. Metab..

[32] Ortega M (2021). Longitudinal investigation of pubertal milestones and hormones as a function of body fat in girls. J. Clin. Endocrinol. Metab..

[33] de Prado-Bert P (2021). The early-life exposome and epigenetic age acceleration in children. Environ. Int..

[34] Jovanovic T (2017). Exposure to violence accelerates epigenetic aging in children. Sci. Rep..

[35] Kaufman J (2018). Adverse childhood experiences, epigenetic measures, and obesity in youth. J. Pediatr..

[36] Galow A, Peleg S (2022). How to slow down the ticking clock: Age-associated epigenetic alterations and related interventions to extend life span. Cells.

[37] Kim C, Harrall K, Glueck D, Dabelea D (2021). Sex steroids and adiposity in a prospective observational cohort of youth. Obes. Sci. Pract..

[38] Aanes H (2023). A new blood based epigenetic age predictor for adolescents and young adults. Sci. Rep..

[39] Hannum G (2013). Genome-wide methylation profiles reveal quantitative views of human aging rates. Mol. Cell.

[40] Horvath S (2013). DNA methylation age of human tissues and cell types. Genome Biol..

[41] Horvath S (2015). Erratum to: DNA methylation age of human tissues and cell types. Genome Biol..

[42] Chen B (2016). DNA methylation-based measures of biological age: Meta-analysis predicting time to death. Aging.

[43] Liu Z (2020). Underlying features of epigenetic aging clocks in vivo and in vitro. Aging Cell.

[44] Brumbaugh D, Crume T, Nadeau K, Scherzinger A, Dabelea D (2012). Intramyocellular lipid is associated with visceral adiposity, markers of insulin resistance, and cardiovascular risk in prepubertal children: The EPOCH study. J. Clin. Endocrinol. Metab..

[45] American Diabetes Association (2020). Classification and diagnosis of diabetes: Standards of medical care in diabetes-2020. Diabetes Care.

[46] Yang I (2018). Epigenetic marks of in utero exposure to gestational diabetes and childhood adiposity outcomes: The EPOCH study. Diabet. Med..

[47] Houseman E (2012). DNA methylation arrays as surrogate measures of cell mixture distribution. BMC Bioinf..

[48] Shiau S (2021). Prenatal gestational diabetes mellitus exposure and accelerated offspring DNA methylation age in early childhood. Epigenetics.

[49] Baird, J., Walker, I., Smith, C. & Inskip, H. *Review of methods for determining pubertal status and age of onset of puberty in cohort and longitudinal studies*, <https://www.closer.ac.uk/wp-content/uploads/CLOSER-resource-Review-of-methods-for-determining-pubertal-status-and-age-of-onset-of-puberty-in-cohort-and-longitudinal-studies.pdf> (2017).

[50] Cole T, Donaldson M, Ben-Schlomo Y (2010). SITAR-a useful instrument for growth curve analysis. Int. J. Epidemiol..

[51] Jaiswal M (2012). Is low birthweight associated with adiposity in contemporary U.S. youth?. J. Dev. Orig. Health Dis..

[52] Marshall W, Tanner J (1968). Growth and physiological development during adolescence. Annu. Rev. Med..

[53] Lamb M, Beers L, Reed-Gillette D, McDowell M (2011). Feasibility of an audio computer-assisted self-interview method to self-assess sexual maturation. J. Adolesc. Health.

[54] Valeri L, Vanderweele TJ (2013). Mediation analysis allowing for exposure-mediator interactions and causal interpretation: Theoretical assumptions and implementation with SAS and SPSS macros. Psychol. Methods.

